# A nomogram for predicting adverse neurovascular events after carotid artery stenting in patients with symptomatic carotid stenosis

**DOI:** 10.3389/fneur.2025.1648838

**Published:** 2025-10-20

**Authors:** Defang Luo, Hailan Zeng, Cheng Xie, Shenhao Xie, Qianliang Huang, Qiuhua Jiang, Mingang Zou

**Affiliations:** ^1^Department of Neurosurgery, Ganzhou People’s Hospital, Ganzhou, Jiangxi, China; ^2^Department of Neurosurgery, The First Affiliated Hospital of Nanchang University, Nanchang, Jiangxi, China

**Keywords:** nomogram, symptomatic carotid artery stenosis, stenting, adverse neurovascular events, predictive model

## Abstract

**Background:**

Carotid artery stenting (CAS) is considered a crucial treatment option for patients with symptomatic carotid artery stenosis. Nevertheless, adverse neurovascular events (ANEs) following this procedure remain a significant challenge. This study aimed to identify risk factors for ANEs and to construct a predictive nomogram to assist in perioperative risk stratification.

**Methods:**

This retrospective study (January 2020–January 2025) enrolled consecutive symptomatic carotid stenosis patients undergoing CAS from two centers: 209 in the training cohort from Ganzhou People’s Hospital and 148 in the external validation cohort from The First Affiliated Hospital of Nanchang University. Patients were categorized into ANE and non-ANE groups based on postoperative outcomes within 30 days. Within the training cohort, independent predictors were identified through a three-step approach: (1) univariate screening, (2) LASSO regression for variable selection, and (3) multivariable logistic regression for final risk factor determination. The nomogram was constructed using R. Internal validation was performed via 1,000 bootstrap resamples. The model’s predictive accuracy and clinical utility were assessed using the C-index, receiver operating characteristic (ROC) curve, and decision curve analysis (DCA).

**Results:**

Age, ulcerated plaque, hemodynamic suppression, and balloon dilation were found to be independent risk factors for the occurrence of ANEs. The Hosmer–Lemeshow test confirmed a good model fit (training: *p* = 0.845; validation: *p* = 0.356), and the calibration curve showed no significant deviation of the predicted probabilities from the actual probabilities. The bootstrap-corrected C-index for internal validation was 0.773. Discriminatory performance was robust, with C-index of 0.802 (training) and 0.816 (validation), and AUCs of 0.798 (95% CI: 0.707–0.889, training) and 0.819 (95% CI: 0.724–0.913, validation). DCA confirmed the substantial clinical value of the nomogram. Furthermore, stratified analyses further revealed different but consistent risk profiles for ischemic and hemorrhagic ANEs, while the composite nomogram maintained robust predictive performance across both subgroups.

**Conclusion:**

The nomogram demonstrated good predictive performance for assessing the risk of ANEs in symptomatic carotid stenosis patients undergoing CAS. Its use aids in optimizing clinical decision-making and reducing postoperative ANEs.

## Introduction

Stroke remains the third leading cause of death and disability globally, with approximately 12 million individuals experiencing their first stroke annually, of which about 62.4% are ischemic strokes (1). Estimates suggest that 20–30% of ischemic strokes originate from carotid atherosclerotic stenosis (2). Carotid artery stenting (CAS), a minimally invasive approach for severe carotid artery stenosis, is an effective alternative to carotid endarterectomy (CEA), particularly for high-risk surgical patients. Although randomized trials such as SPACE-2 have questioned the efficacy of CAS in asymptomatic patients, current guidelines continue to endorse its use in individuals with symptomatic carotid stenosis of ≥50%—the population investigated in this study (3). In clinical practice, even with the assistance of embolic protection devices (EPDs) and stent renewal, adverse neurovascular events (ANEs), such as transient ischemic attack (TIA), ischemic or hemorrhagic stroke, and cerebral hyperperfusion syndrome (CHS), continue to occur post-CAS (4, 5). While ischemic events are more prevalent, CHS—characterized by impaired cerebral autoregulation and increased ipsilateral cerebral blood flow—represents a serious but often underrecognized complication. All these events may lead to neurological deterioration, prolonged hospitalization, or even mortality. Notably, they appear to share overlapping risk factors, including advanced age, high-grade stenosis, and perioperative blood pressure instability (6–8). Therefore, the early and effective identification of individuals at high risk for CAS complications among patients with carotid stenosis remains critical.

Several recent studies have explored predictors of post-CAS complications, but most have focused on isolated outcomes. For example, one carotid ultrasound study found that the jellyfish sign, proximal calcification, and elevated LDL cholesterol were predictive of new diffusion-weighted imaging (DWI) lesions after CAS (9). Another machine learning–based model using XGBoost identified internal carotid artery peak systolic velocity, LDL cholesterol, and procedure type (CEA vs. CAS) as key predictors of early ischemic events (10). Research on CHS is relatively limited; a transcranial Doppler–based study demonstrated that specific hemodynamic parameters could predict CHS risk, highlighting the role of cerebral autoregulation (11). Although TIA, stroke, and CHS differ in pathophysiology, they may share a common perioperative risk profile. However, existing models remain fragmented and focus on specific complications. This lack of integration limits their clinical value in comprehensive risk stratification. To date, no validated tool is available for predicting the overall risk of ANEs in patients with symptomatic carotid stenosis undergoing CAS.

This study constructed and validated a nomogram for predicting ANE risk by comprehensively analyzing preoperative demographic, imaging, and intraoperative technical data from patients undergoing CAS. This tool offers clinicians a practical and individualized risk stratification approach, potentially reducing the incidence of postoperative neurovascular complications.

## Methods

### Patient population

Between January 2020 and January 2025, clinical data were retrospectively collected from 679 patients with carotid stenosis who underwent stenting procedures at Ganzhou People’s Hospital and The First Affiliated Hospital of Nanchang University. The inclusion criteria were as follows: (1) A confirmed diagnosis of symptomatic carotid artery stenosis, as evidenced by a history of cerebral infarction or symptoms of TIA within the past 6 months; (2) Carotid artery stenosis ≥ 50%; and (3) Initial CAS treatment. The exclusion criteria were as follows: (1) Carotid artery stenosis caused by non-atherosclerotic conditions such as dissection or large-artery inflammation; (2) Patients whose clinical or imaging data were incomplete; (3) Previous CEA or CAS intervention on the target carotid artery; (4) Patients undergoing emergency CAS for acute large-vessel occlusion; and (5) Severe coagulation disorders, hepatic or renal insufficiency, and psychiatric disorders, among other conditions. This study was reviewed by the Ethics Committee of Ganzhou People’s Hospital (No. Ky2024018). Written informed consent was waived as the retrospective study involved only the analysis of previously collected data.

### Data collection and definition

Patient demographics and radiology and surgical records were obtained from the hospital’s medical records system. The patient demographic data included age, sex, hypertensive disease status, diabetes mellitus status, hyperlipidemia status, coronary cardiopathy status, and smoking history prior to the procedure. CYP2C19 genotyping results were retrieved and stratified into three clopidogrel resistance categories: no resistance (^*^1/^*^1, ^*^1/^*^17, ^*^17/^*^17), moderate resistance (^*^1/^*^2, ^*^1/^*^3, ^*^2/^*^17, ^*^3/^*^17), and severe resistance (^*^2/^*^2, ^*^2/^*^3, ^*^3/^*^3) (12). Additionally, radiographic data on the rate of carotid stenosis, as well as the length, thickness, morphology (regular or irregular), and characteristics (calcified or ulcerated) of the plaques, were acquired. The carotid stenosis percentage was calculated as (1 – the ratio of the narrowest diameter to the original diameter) × 100% (13). Plaque length was measured as the total distance between the top and bottom of each lesion, while plaque thickness referred to the maximum thickness of the plaque in an axial view. Based on preoperative cervical vascular computed tomography angiography (CTA), the atherosclerotic plaque morphology was classified as follows: plaques with a computed tomography (CT) value of ≥120 Hounsfield units (Hu) were categorized as calcified, while those with a CT value below this threshold were considered non-calcified. Plaques were classified as ulcerative if the spread of the contrast agent along the inner surface of the arterial plaque exceeded 1 mm (14). The procedure adhered to the published guideline protocol (15). Intraoperative data included blood pressure variability (BPV), aortic arch typing, carotid tortuosity, hemodynamic suppression, use of balloon dilation, and stent type. BPV was quantified as the coefficient of variation, calculated by dividing the standard deviation of systolic pressure measurements by the mean systolic pressure and expressed as a percentage (16). The type of aortic arch was determined by comparing the vertical distance from the top of the aortic arch to the origin of the innominate artery (a) with the diameter of the left common carotid artery, (b) type I: a/b < 1, type II: 1 ≤ a/b < 2, type III: a/b ≥ 2 (17). Carotid artery tortuosity was identified by the presence of “C” type curvature, “O” type coiling, or “S” type twisting (18). Hemodynamic suppression was diagnosed if the patient exhibited either asymptomatic or symptomatic hypotension (systolic blood pressure below 90 mmHg) or bradycardia (heart rate lower than 60 beats per minute) intraoperatively or postoperatively (19). Prophylactic use of temporary cardiac pacemakers, with a pacing rate of 60 beats per minute, was considered indicative of hemodynamic depression if the intraoperative or postoperative electrocardiogram displayed a clear pacing signal. Patients underwent head CT and MRI scans between 24 and 72 h after CAS.

Patients were categorized into ANE and non-ANE groups according to the presence or absence of ANEs during the 30-day follow-up period. The ANEs included episodes of TIA, ischemic or hemorrhagic stroke, and CHS. TIA was defined as a transient episode of neurological dysfunction caused by focal brain, spinal cord, or retinal ischemia, without acute cerebral infarction (20). To minimize the risk of TIA misclassification based solely on medical record documentation, a standardized adjudication process was adopted: (a) two neurologists independently and blindly reviewed all suspected TIA cases; (b) the initial “TIA vs. non-TIA” judgments from each neurologist were recorded separately; and (c) in cases of disagreement, a third senior neurologist provided the final adjudication. Inter-rater agreement was assessed using Cohen’s kappa statistic, with detailed results reported in the Supplementary Table S1. Hemorrhagic stroke events were characterized by the presence of new punctate or confluent high-density shadows indicative of blood on head CT scans. Ischemic stroke events were identified by new high-signal areas on MRI scans, particularly on DWI sequences, accompanied by neurological deficits corresponding to the lesion. CHS was defined by (a) new-onset neurological symptoms post-CAS (e.g., severe headache, focal neurological deficits, seizures, or impaired consciousness), (b) exclusion of new cerebral ischemic lesions on postoperative neuroimaging, and (c) and objective evidence of cerebral hyperperfusion, defined as either: a > 100% increase in ipsilateral middle cerebral artery flow velocity versus preoperative baseline, or a > 100% increase in cerebral blood flow (CTP/MR perfusion) versus contralateral hemisphere (21, 22). Although the composite endpoint of ANEs provides overall statistical power, it potentially masks crucial heterogeneity in underlying pathophysiology. To ascertain whether predictors exert uniform effects across all event types, and to identify distinct risk factor profiles specific to each pathological pathway, we performed a pre-specified subgroup analysis. To this end, ANEs were categorized into two groups based on their distinct pathophysiologies: (a) Ischemic ANEs, comprising TIA and ischemic stroke, based on their shared etiology of thromboembolism or hemodynamic insufficiency leading to cerebral ischemia (23, 24). (b) Hemorrhagic ANEs, comprising hemorrhagic stroke and CHS, based on their shared pathophysiology of disruption of the blood–brain barrier and extravasation of blood, either due to vessel rupture or from overwhelmed cerebral autoregulation and capillary leakage (25, 26).

### Statistical analysis

Categorical variables are expressed herein as frequencies (percentages) and were compared using the *χ*^2^ test. Continuous variables with a normal distribution are reported as the mean ± standard deviation and were analyzed using Student’s *t*-test, while variables with skewed distributions are presented as the median (interquartile range) and were assessed using the Wilcoxon test. Missing covariates were handled via multiple imputation (27). Variable selection employed a sequential approach: univariate screening (*p* < 0.1) identified candidate predictors, followed by least absolute shrinkage and selection operator (LASSO) regression with sevenfold cross-validation (*λ* determined by minimizing binomial deviance). Variables excluded by LASSO but clinically critical were retained based on established relevance. Continuous variables were tested for nonlinearity using restricted cubic splines. The number of knots was set at four, a choice guided by the Akaike information criterion to balance model fit and complexity, consistent with established approaches (28). The selected candidate variables were then incorporated into the multiple logistic regression analysis, from which statistically significant variables were utilized to construct a personalized nomogram for predicting ANEs. Bayesian validation with weakly informative priors was conducted to evaluate predictor robustness under EPV = 8 constraints. The model’s discriminative ability was evaluated using the concordance index (C-index), where a value > 0.7 indicates favorable accuracy. The consistency and predictive accuracy of the model were measured by plotting calibration curves and the area under the receiver operating characteristic (ROC) curve (AUC). The fit of the model was evaluated by the Hosmer–Lemeshow test, while its net benefit was estimated through decision curve analysis (DCA). The corrected C-index was obtained by bootstrapping (1,000 resamplings) to internally validate the predictive model. To further evaluate whether predictors exert uniform effects across different event types, we conducted a pre-specified subgroup analysis. Given the limited number of events within each subgroup, we applied Firth’s penalized logistic regression to mitigate small-sample bias and obtain more stable estimates of odds ratios and confidence intervals. For model interpretability, we further used the SHAP (SHapley Additive exPlanations) framework to quantify the contribution of each predictor to the model’s output. Considering that event numbers declined substantially after stratification, we did not rebuild independent models for each subgroup. Instead, we applied the predictors identified in the composite model to the ischemic and hemorrhagic subgroups, and assessed their discriminatory performance using ROC curve analysis. Statistical processing and graphing were conducted using SPSS (version 21.0) and RStudio (version 4.3.3). A *p*-value of < 0.05 was considered to indicate statistical significance.

## Results

### Baseline characteristics and predictor screening

**Figure 1:**
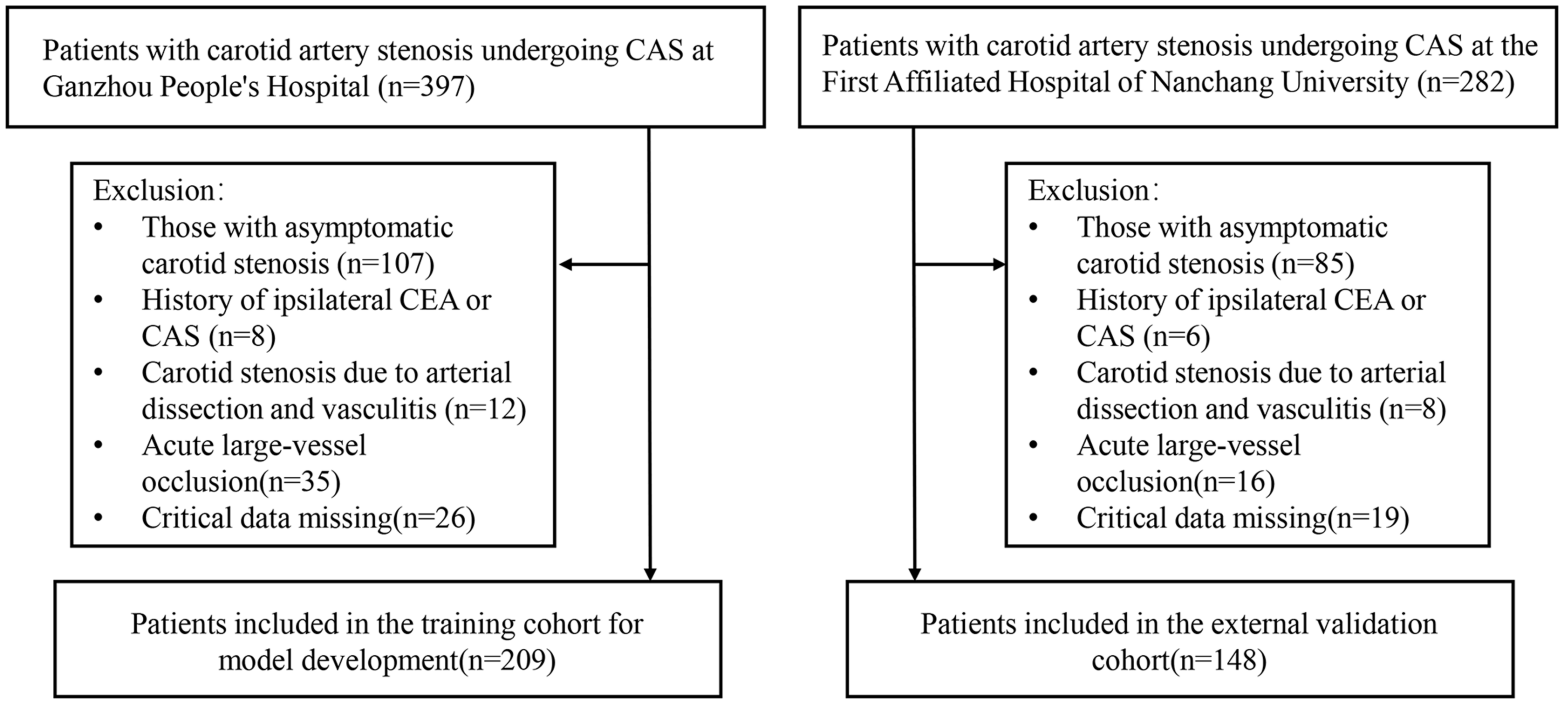
Flowchart for patient screening.

**Figure 2:**
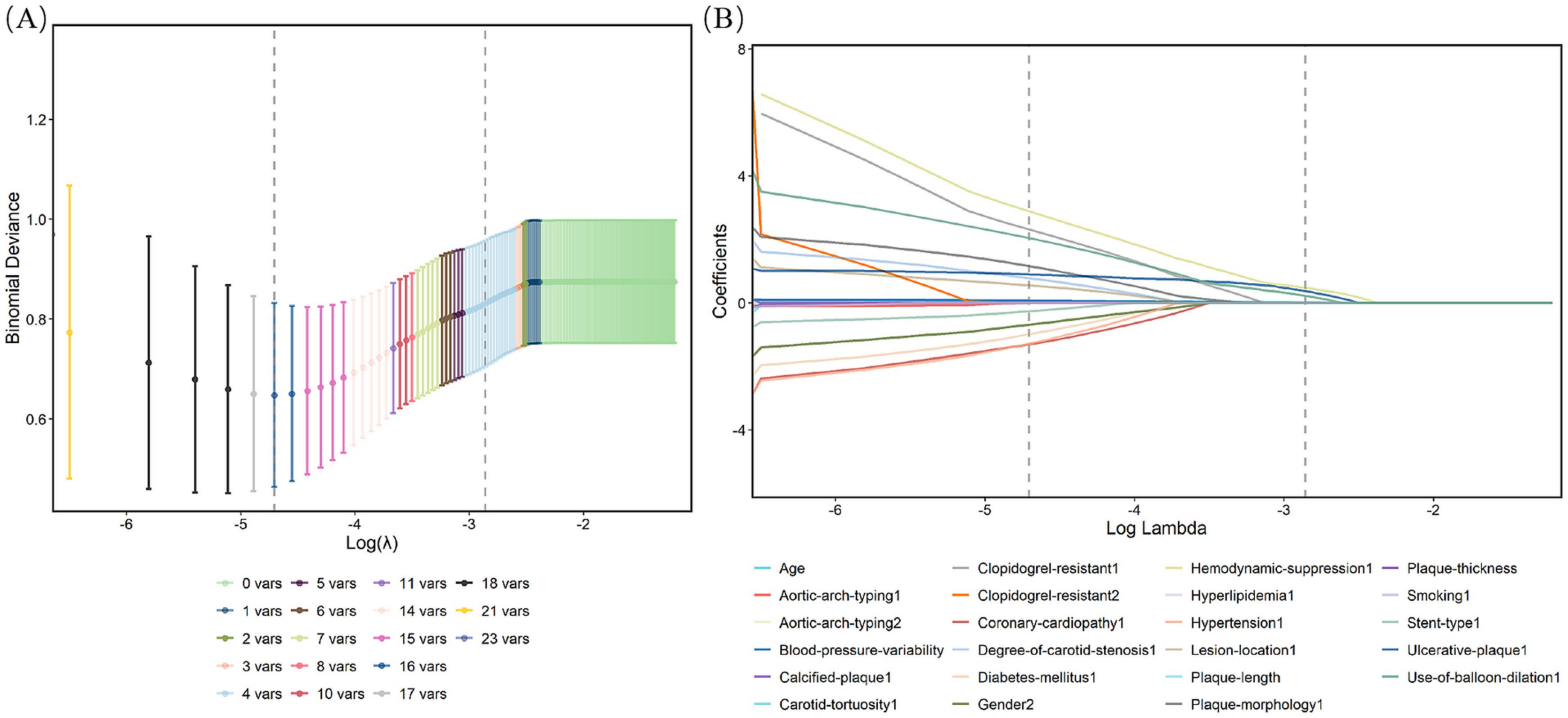
Characteristic factors were screened by the LASSO binary logistic regression model. **(A)** Log(lambda) values for the 21 features in the LASSO analysis. A coefficient distribution plot was generated from the log(lambda) sequence. **(B)** LASSO model parameter selection was conducted through sevenfold cross-validation. Four nonzero coefficients were identified by determining the optimal lambda values using the 1 standard error (1-SE) criterion. Vertical lines were plotted at the points corresponding to the minimum and 1-SE standard.

This study enrolled 209 patients from Ganzhou People’s Hospital (training cohort) and 148 patients from The First Affiliated Hospital of Nanchang University (validation cohort), all of whom met the inclusion and exclusion criteria (Figure 1). Baseline demographic and clinical features of both cohorts are summarized in Table 1, with no significant differences (*p* > 0.05). Within the training cohort, 32 patients (15.31%) experienced ANEs during follow-up, comprising 35 distinct clinical events. The cohort comprised: isolated TIA in 10 cases (4.78%), isolated symptomatic cerebral infarction in 7 cases (3.35%), coexisting TIA and symptomatic cerebral infarction in 2 cases (0.96%), isolated intracranial hemorrhage in 3 cases (1.44%), CHS progressing to intracranial hemorrhage in 1 case (0.48%), and isolated CHS in 9 cases (4.31%). Univariate analysis identified seven candidate predictors associated with ANE: age, degree of carotid stenosis, plaque morphology, ulcerated plaque, hemodynamic suppression, blood pressure variability, and use of balloon dilation (Table 2). LASSO regression assigned nonzero coefficients to four variables: age, ulcerated plaque, hemodynamic suppression, use of balloon dilation (Figure 2). Given the potential clinical relevance of carotid stenosis degree, plaque morphology, and blood pressure variability to neurovascular outcomes, we conservatively retained all seven predictors in multivariate modeling. LASSO’s coefficient shrinkage still optimized model stability by resolving multicollinearity.

**Table 1:** Baseline characteristics in the training cohort and validation cohort.

Variables	Training cohort (*n* = 209)	Validation cohort (*n* = 148)	*p*-value
Age (years), mean ± SD	65.25 ± 6.95	64.76 ± 6.47	0.494
Gender, *n* (%)			0.713
Male	172 (82.3)	124 (83.8)	
Female	37 (17.7)	24 (16.2)	
Hypertension, *n* (%)	157 (75.1)	105 (70.9)	0.379
Diabetes mellitus, *n* (%)	57 (27.3)	53 (35.8)	0.085
Hyperlipidemia, *n* (%)	70 (33.5)	51 (34.5)	0.849
Coronary cardiopathy, *n* (%)	42 (20.1)	32 (21.6)	0.726
Smoking, *n* (%)	123 (58.9)	90 (60.8)	0.710
Clopidogrel resistant, *n* (%)			0.994
None	74(39.6)	53(40.2)	
Moderate	79(42.2)	55(41.7)	
Severe	34 (18.2)	24 (18.2)	
Lesion location, *n* (%)			0.685
Internal carotid artery	182 (87.1)	131 (88.5)	
Common carotid artery	27 (12.9)	17 (11.5)	
Degree of carotid stenosis, *n* (%)			0.652
<70%	77 (36.8)	58 (39.2)	
≥70%	132 (63.2)	90 (60.8)	
Plaque length (mm), mean ± SD	20.32 ± 6.74	20.49 ± 6.82	0.815
Plaque thickness (mm), mean ± SD	3.80 ± 0.78	3.74 ± 0.75	0.479
Plaque morphology, *n* (%)			0.901
Regular	120 (57.4)	84 (56.8)	
Irregular	89 (42.6)	64 (43.2)	
Plaque characteristics, *n* (%)			
Calcified plaque	73 (34.9)	58 (39.2)	0.411
Ulcerated plaque	50 (23.9)	34 (23.0)	0.835
Aortic arch typing, *n* (%)			0.709
I	59 (28.2)	36 (24.3)	
II	124 (59.3)	92 (62.2)	
III	26 (12.4)	20 (13.5)	
Carotid tortuosity, *n* (%)	113 (54.1)	83 (56.1)	0.706
Hemodynamic suppression, *n* (%)	76 (36.4)	50 (33.8)	0.615
Blood pressure variability (%), mean ± SD	14.14 ± 4.50	13.99 ± 3.80	0.741
Use of balloon dilation, *n* (%)	52 (24.9)	41 (27.7)	0.549
Stent type, *n* (%)			0.771
Closed-loop	55 (26.3)	41 (27.7)	
Open-loop	154 (73.7)	107 (72.3)	

**Table 2:** Univariate analysis in the training cohort.

Variables	ANE group (*n* = 32)	Non-ANE group (*n* = 177)	OR (95%CI)	*p*-value
Age (years), mean ± SD	68.94 ± 6.48	64.59 ± 6.84	1.095 (1.035–1.158)	0.002
Gender, *n* (%)				
Male	27 (84.4)	145 (81.9)	0.839 (0.300–2.346)	0.738
Female	5 (15.6)	32 (18.1)		
Hypertension, *n* (%)	25 (78.1)	132 (74.6)	1.218 (0.493–3.006)	0.670
Diabetes mellitus, *n* (%)	7 (21.9)	50 (28.2)	0.711 (0.289–1.749)	0.458
Hyperlipidemia, *n* (%)	12 (37.5)	58 (32.8)	1.231 (0.563–2.689)	0.602
Coronary cardiopathy, *n* (%)	7 (21.9)	35 (19.8)	1.136 (0.454–2.840)	0.785
Smoking, *n* (%)	20 (62.5)	103 (58.2)	1.197 (0.551–2.600)	0.649
Clopidogrel resistant, *n* (%)				
None	13(40.6)	66(37.3)		
Moderate	16(50.0)	75(42.4)	1.083 (0.485–2.418)	0.846
Severe	3(9.4)	36(20.3)	0.423 (0.113–1.583)	0.201
Lesion location, *n* (%)				
Internal carotid artery	26 (81.2)	156 (88.1)	1.714 (0.632–4.650)	0.290
Common carotid artery	6 (18.8)	21 (11.9)		
Degree of carotid stenosis, *n* (%)				
<70%	6 (18.8)	71 (40.1)	2.903 (1.137–7.410)	0.026
≥70%	26 (81.2)	106 (59.9)		
Plaque length (mm), mean ± SD	19.86 ± 6.43	20.40 ± 6.80	0.988 (0.933–1.046)	0.674
Plaque thickness (mm), mean ± SD	3.96 ± 0.81	3.77 ± 0.77	1.366 (0.844–2.212)	0.204
Plaque morphology, *n* (%)				
Regular	14 (43.8)	106 (59.9)	1.920 (0.897–4.106)	0.093
Irregular	18 (56.2)	71 (40.1)		
Plaque characteristics, *n* (%)				
Calcified plaque	15 (46.9)	58 (32.8)	1.810 (0.845–3.879)	0.127
Ulcerated plaque	15 (46.9)	35 (19.8)	3.580 (1.630–7.862)	<0.001
Aortic arch typing, *n* (%)				
I	8 (25.0)	51 (28.8)		
II	17 (53.1)	107 (60.5)	1.013 (0.410–2.501)	0.978
III	7 (21.9)	19 (10.7)	2.349 (0.749–7.366)	0.143
Carotid tortuosity, *n* (%)	20 (62.5)	93 (52.5)	1.505 (0.694–3.265)	0.300
Hemodynamic suppression, *n* (%)	21 (65.6)	55 (31.1)	4.235 (1.911–9.386)	<0.001
Blood pressure variability (%), mean ± SD	15.85 ± 4.17	13.83 ± 4.50	1.110 (1.016–1.213)	0.021
Use of balloon dilation, *n* (%)	15 (46.9)	37 (20.9)	3.339 (1.526–7.306)	0.003
Stent type, *n* (%)				
Closed-loop	10 (31.2)	45 (25.4)	0.750 (0.330–1.704)	0.492
Open-loop	22 (68.8)	132 (74.6)		

ANE, adverse neurovascular event.

### Multivariable analysis and nomogram development

**Figure 3:**
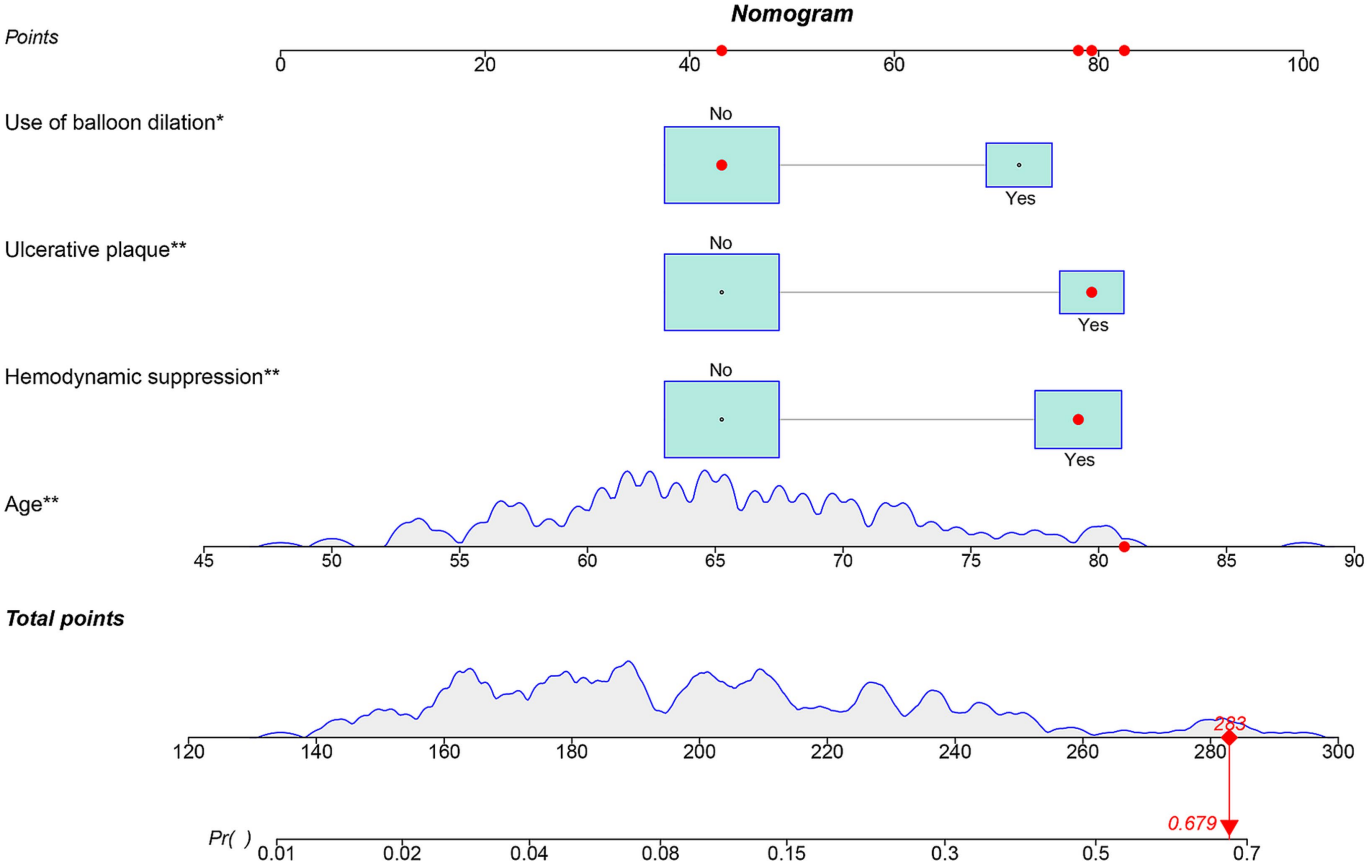
Nomogram for predicting the occurrence of ANEs after CAS for symptomatic carotid stenosis.

Independent risk factors for ANE post-CAS were established through multivariable logistic regression, namely, age (OR: 1.095, 95% CI: 1.026–1.169, *p* = 0.007), ulcerated plaque (OR: 2.986, 95% CI: 1.203–7.414, *p* = 0.018), hemodynamic suppression (OR: 2.904, 95% CI: 1.118–7.543, *p* = 0.029), and the use of balloon dilation (OR: 3.445, 95% CI: 1.360–8.728, *p* = 0.009) (Table 3). Age was modeled as a continuous linear term; restricted cubic spline analysis detected no significant nonlinearity (nonlinearity test *p* = 0.063), supporting its linear specification in the final model (Supplementary Figure S1). To further validate these predictors, Bayesian posterior distribution plots were generated. All variables exhibited clear unimodal distributions with 95% credible intervals excluding zero. Notably, the posterior means provided a ranking of variable contributions that was fully aligned with the direction of effects observed in the traditional logistic regression analysis (Supplementary Figures S2–S5). The optimal cutoff value for age was determined to be 64.5 years based on the ROC curve (Supplementary Figure S6). To assess the risk of ANEs following CAS, the significant factors identified were incorporated to construct a nomogram (Figure 3). As illustrated, each predictive variable was allocated a score, and the cumulative scores were then calculated to estimate the likelihood of ANE occurrence.

**Table 3:** Multivariate logistic regression analysis in the training cohort.

Variables	*β*	SE	OR (95%CI)	*p*-value
Age	0.091	0.033	1.095 (1.026–1.169)	0.007
Degree of carotid stenosis	0.759	0.588	2.135 (0.675–6.756)	0.197
Plaque morphology	0.765	0.456	2.150 (0.879–5.256)	0.093
Ulcerated plaque	1.094	0.464	2.986 (1.203–7.414)	0.018
Hemodynamic suppression	1.066	0.487	2.904 (1.118–7.543)	0.029
Blood pressure variability	0.095	0.054	1.099 (0.988–1.223)	0.081
Use of balloon dilation	1.237	0.474	3.445 (1.360–8.728)	0.009

### Validation of the nomogram for ANE occurrence

**Figure 4:**
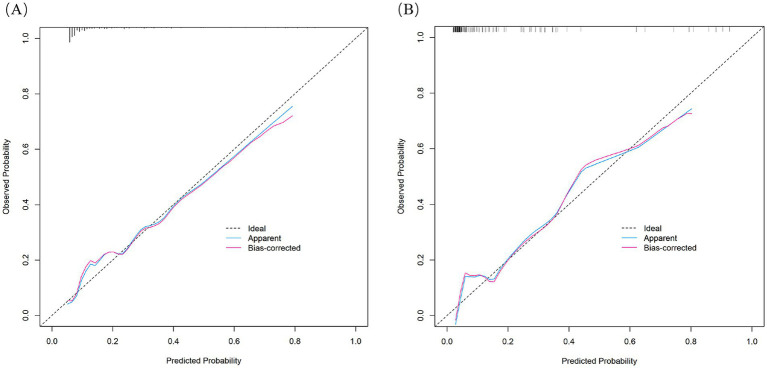
Calibration curves of the nomogram in the training cohort **(A)** and validation cohort **(B)**.

**Figure 5:**
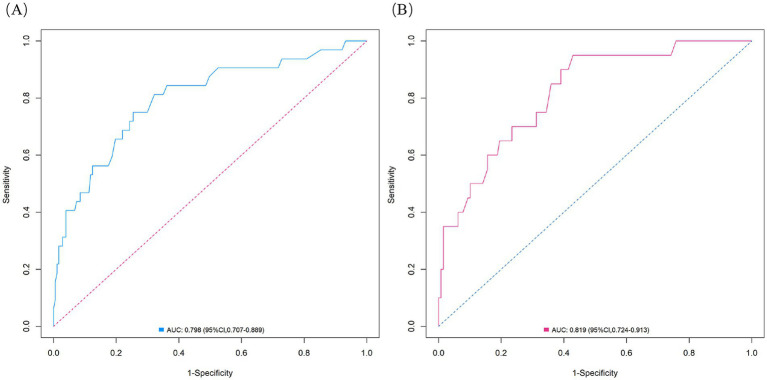
ROC curves for the ANE nomogram in the training cohort **(A)** and validation cohort **(B)**.

**Figure 6:**
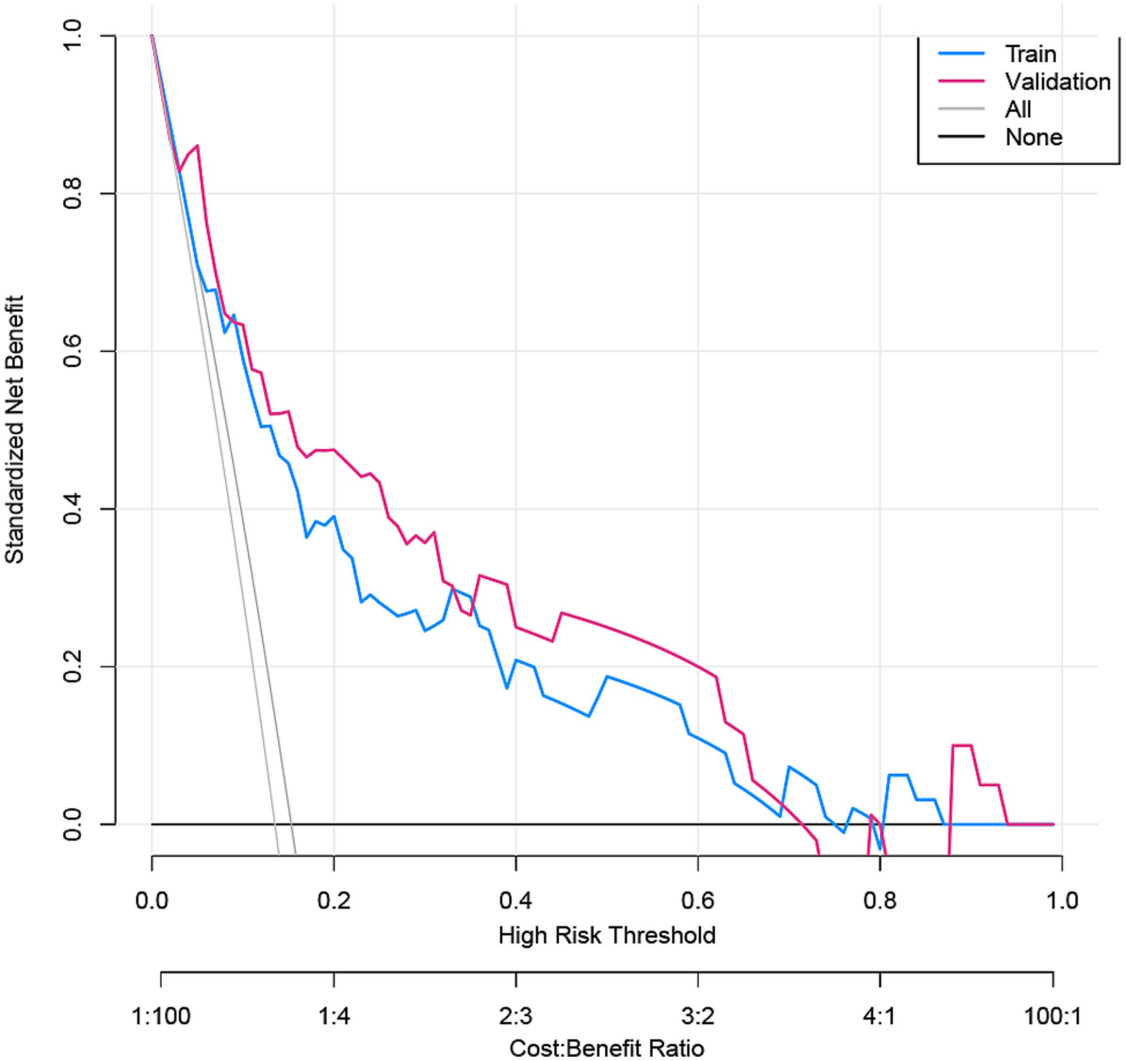
DCA for the ANE nomogram in the training cohort and validation cohort.

The nomogram demonstrated substantial predictive accuracy, achieving a C-index of 0.802 in the training cohort and 0.816 in the validation cohort. Internal bootstrap validation (1,000 resamplings) on the training cohort yielded a corrected C-index of 0.773, indicating favorable predictive performance and robustness. Hosmer–Lemeshow testing showed no significant evidence of misfit (training: *χ*^2^ = 4.135, *p* = 0.845; validation: *χ*^2^ = 8.843, *p* = 0.356). The calibration curve further demonstrated close agreement between the nomogram-predicted probabilities of ANEs after CAS and actual outcomes (Figure 4). Furthermore, to evaluate and correct for any potential over optimism in the training set, we generated a bootstrap-corrected calibration curve using the training cohort (Supplementary Figure S7), which further confirmed the model’s robust calibration performance. Additionally, the model exhibited excellent discriminative ability, with AUC values of 0.798 (95% CI: 0.707–0.889) for the training cohort and 0.819 (95% CI: 0.724–0.913) for the validation cohort (Figure 5). DCA revealed that employing the nomogram provided a net clinical benefit over the no intervention strategy across probability thresholds ranging from 0.07 to 0.75 (training) and 0.04 to 0.72 (validation) (Figure 6).

### Stratified analysis of ischemic and hemorrhagic ANEs

**Figure 7:**
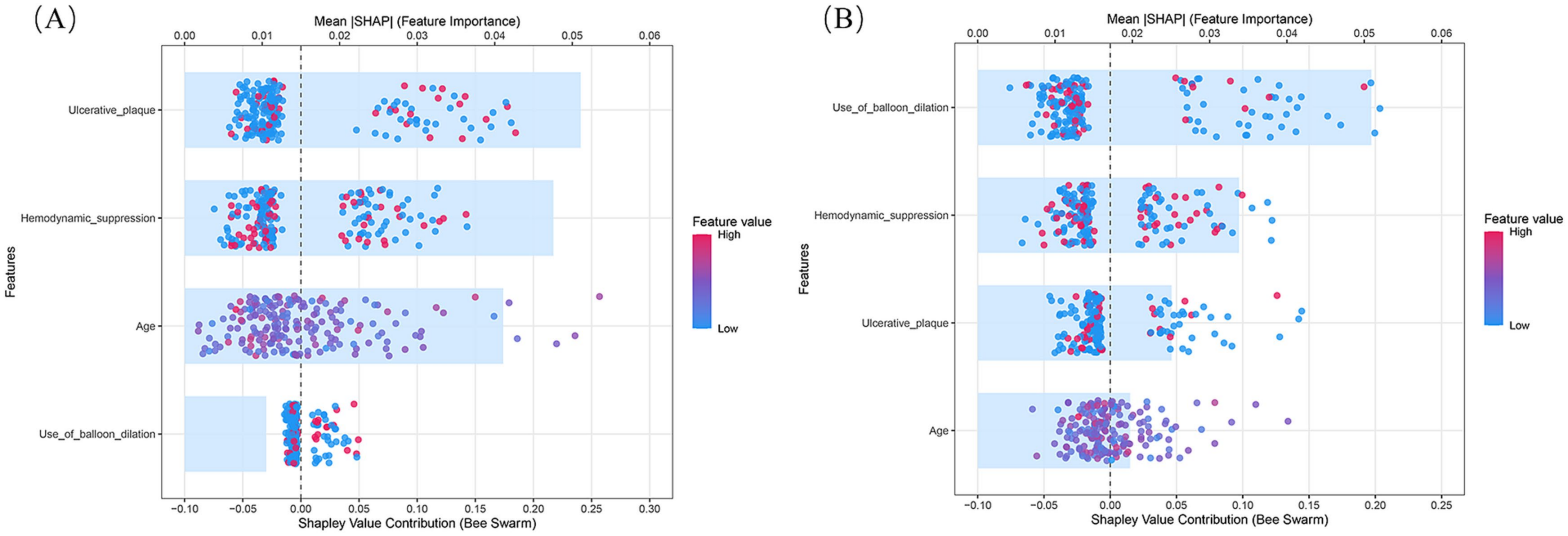
SHAP summary plots of the composite prediction model stratified by outcome subtype. **(A)** Ischemic ANEs subgroup; **(B)** Hemorrhagic ANEs subgroup.

**Figure 8:**
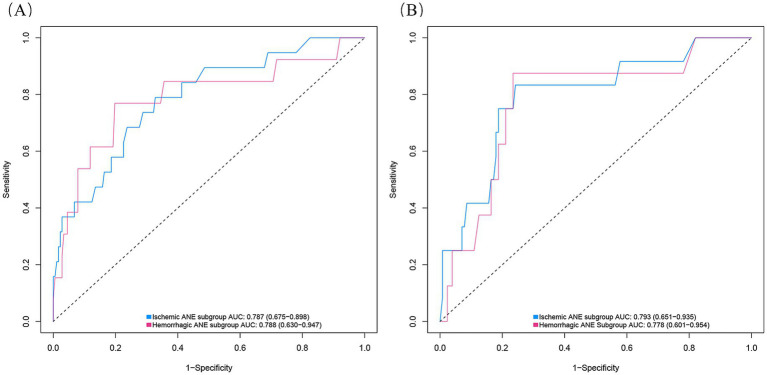
ROC curves of the composite model stratified by ANE subtype in the training cohort **(A)** and validation cohort **(B)**.

We further performed stratified analyses to separately evaluate ischemic and hemorrhagic ANEs. In the training cohort, Firth logistic regression identified Age (OR = 1.096, 95% CI 1.020–1.183, *p* = 0.013), Ulcerated plaque (OR = 4.268, 95% CI 1.570–11.844, *p* = 0.005), and Hemodynamic suppression (OR = 3.313, 95% CI 1.204–9.685, *p* = 0.020) as independent risk factors for ischemic ANEs (Table 4). By contrast, hemorrhagic ANEs were independently associated with Hemodynamic suppression (OR = 3.520, 95% CI 1.023–13.681, *p* = 0.046), Blood pressure variability (OR = 1.163, 95% CI 1.006–1.369, *p* = 0.041), and Use of balloon dilation (OR = 5.943, 95% CI 1.790–22.341, *p* = 0.004) (Table 5). In the validation cohort, results exhibited trends consistent with those observed in the training set (Supplementary Tables S2, S3). SHAP analysis further delineated the distinct contributions of predictors within each subgroup (Figure 7). In ischemic ANEs, Ulcerated plaque, Hemodynamic suppression, and Age exerted the greatest impact, whereas in hemorrhagic ANEs, Balloon dilation and Hemodynamic suppression emerged as dominant contributors. Notably, the color gradient in the bee swarm plots indicated that increasing Age was associated with elevated risk of both ischemic and hemorrhagic events. After stratification, the composite model retained acceptable predictive performance. In the training cohort, AUCs were 0.787 for ischemic and 0.788 for hemorrhagic ANEs (Figure 8A); in the validation cohort, AUCs were 0.793 and 0.778, respectively (Figure 8B). Compared with the original composite model, stratified models demonstrated slightly lower discrimination, yet remained within clinically meaningful ranges.

**Table 4:** Univariate and multivariate Firth penalized logistic regression analyses for ischemic ANEs in the training cohort.

Variables	Univariate analysis (Ischemic ANEs)		Multivariate analysis (Ischemic ANEs)	
	OR (95%CI)	*p*-value	OR (95%CI)	*p*-value
Age	1.100 (1.027–1.177)	0.006	1.102 (1.022–1.192)	0.013
Ulcerated plaque	4.508 (1.703–11.934)	0.002	4.573 (1.617–13.282)	0.004
Hemodynamic suppression	3.803 (1.420–10.183)	0.008	3.576 (1.248–10.979)	0.020
Use of balloon dilation	1.746 (0.622–4.906)	0.290	1.456 (0.427–4.537)	0.528

ANE, adverse neurovascular event.

**Table 5:** Univariate and multivariate Firth penalized logistic regression analyses for hemorrhagic ANEs in the training cohort.

Variables	Univariate analysis (Hemorrhagic ANEs)		Multivariate analysis (Hemorrhagic ANEs)	
	OR (95%CI)	*p*-value	OR (95%CI)	*p*-value
Age	1.081 (0.998–1.172)	0.057	1.068 (0.967–1.180)	0.190
Ulcerated plaque	2.536 (0.782–8.227)	0.121	2.552 (0.591–10.580)	0.193
Hemodynamic suppression	4.991 (1.473–16.906)	0.010	4.060 (1.082–17.768)	0.044
Blood pressure variability	1.198 (1.039–1.382)	0.013	1.182 (1.013–1.407)	0.043
Use of balloon dilation	8.514 (2.483–29.193)	<0.001	7.151 (1.976–30.414)	0.004

ANE, adverse neurovascular event.

## Discussion

Although carotid artery stenting decreases the risk of future ischemic stroke in patients with symptomatic carotid artery stenosis by reshaping vascular structure and restoring blood flow (29), ANEs such as TIA, stroke, and cognitive impairment continue to pose challenges in clinical practice. Consequently, it is essential to analyze the factors associated with the occurrence of ANEs following CAS and to establish relevant predictive models. In this study, the incidence of ANEs within 30 days after CAS for symptomatic carotid artery stenosis was 15.31%, with rates of 5.74% for TIA and 6.22% for stroke, consistent with previous literature (30). However, the incidence of CHS in this cohort was 4.78%, falling within the 2.2–11.2% range reported in prior studies (8, 11), yet reflecting notable variability. This discrepancy may stem from several factors, including differences in patient selection criteria, perioperative blood pressure control protocols, imaging surveillance intensity, and diagnostic definitions of CHS. For instance, some studies include only clinically overt CHS, while others capture subclinical cases detected via transcranial Doppler or perfusion imaging. In this study, 21 variables potentially associated with postoperative ANEs were analyzed among a training cohort of 209 patients undergoing CAS for symptomatic carotid stenosis. Age, ulcerated plaque, hemodynamic suppression, and the use of balloon dilation were identified as independent risk factors for ANEs through univariate analysis, LASSO, and multivariable logistic regression analysis. On this basis, a risk prediction nomogram was successfully developed, providing quantifiable probabilities and describing each predictor’s contribution to the risk of ANEs after CAS.

Consistent with previous reports (6, 31), age was confirmed as an independent risk factor for ANEs following CAS in this study (OR = 1.095). This indicates that the risk of ANEs increases by an average of 9.5% with each additional year of age. The extensive atherosclerosis, abnormal or tortuous arterial anatomy, and complex plaques observed in elderly patients not only complicate the CAS procedure but also increase the risk of plaque detachment or vascular injury. In addition, the presence of multiple coexisting comorbidities may lead to increased stroke risk in these patients post-CAS (32, 33). Moreover, advancing age was associated with diminished cerebral autoregulatory capacity and increased blood–brain barrier permeability, which may have elevated the risk of postoperative CHS and intracerebral hemorrhage (34, 35). Additionally, age-related microvascular fragility and reduced cerebrovascular reactivity may have increased sensitivity to intraoperative microemboli. These emboli could have induced vasoreactive changes or caused distal vessel occlusion, leading to reduced perfusion in functionally critical brain regions and thereby increasing the risk of postoperative TIA or ischemic stroke (36, 37). In this study, the optimal cutoff value for age was determined by receiver operating characteristic (ROC) curve analysis to be 64.5 years, which suggests that individuals above this age are at an increased risk of ANEs when undergoing CAS. Thus, enhanced monitoring and precautions may be necessary for older patients in the perioperative period of CAS.

This study also revealed that the presence of ulcerated plaques was the strongest predictor of ANE. Patients with ulcerated plaques had an approximately 3.0-fold greater risk of developing ANE after CAS than did patients without such plaques. More stable plaques were associated with thick, unruptured fibrous caps. In contrast, vulnerable or ruptured fibrous caps, related to ulceration, are crucial determinants of ischemic stroke. Beyond macroscopic structure, the profound vulnerability of ulcerated plaques may be driven by an active intraplaque microenvironment involving processes such as inflammation and ferroptosis (38). This inherent fragility implies that the irregular surface readily serves as an attachment point for platelet aggregation; concurrently, reverse blood flow within the ulcer niche further increases the risk of thrombus formation. Irregular ulcerated plaques readily serve as attachment points for platelet aggregation, while reverse blood flow within ulcer niches further increases the risk of thrombus formation (39, 40). As the procedure progresses, the distal EPD can potentially damage lesions, particularly ulcerated plaques, as the guidewire passes through the stenotic area. There is also a risk of emboli overflow during device retrieval (41). Previous studies have reported that fragments from vulnerable ulcerated plaques are finer, and the microemboli from these fragments are less likely to be effectively filtered by distal protection devices. These microemboli can obstruct small cerebral vessels, increasing the risk of postoperative TIA or ischemic stroke (42). In identifying ulcerated plaques, Saba et al. (43) found that the majority of carotid plaque ulcerations diagnosed via color Doppler ultrasound were overlooked, while CTA exhibited a high sensitivity of 93.75% for detecting ulcerations. Therefore, in clinical practice, the importance of CTA as an essential preprocedural assessment tool for CAS should be emphasized, with attention given to the characteristics of plaques, especially those that are ulcerated.

Hemodynamic suppression and the use of balloon dilation have also been identified as independent risk factors for ANEs following CAS. Perioperative hemodynamic suppression is closely associated with the number of new ischemic lesions identified by DWI, while insufficient perfusion increases the susceptibility of the brain to embolism (44). During carotid revascularization surgery, a surge in blood flow stimulates the carotid sinus, affecting the sinoatrial and atrioventricular nodes and increasing cardiac vagal tone, potentially leading to bradycardia and a decrease in peripheral vascular resistance, which may result in hypotensive episodes (45). These hemodynamic alterations may cause insufficient perfusion in vulnerable brain regions, increasing the risk of postoperative TIA or ischemic stroke (46). Therefore, it is essential to monitor and regulate perioperative hemodynamics, especially in patients with baseline bradycardia. Preventive strategies, such as prophylactic administration of atropine or the use of temporary pacemakers, are recommended. In addition, patients with chronic cerebral hypoperfusion may have impaired autoregulatory capacity, making them more susceptible to CHS after stent placement (47). In such cases, an inability of cerebral vessels to constrict in response to restored perfusion could result in microvascular leakage, vasogenic edema, or intracerebral hemorrhage. Therefore, strict control of perioperative blood pressure fluctuations is essential to reduce the risk of CHS. Postoperative continuous blood pressure monitoring and individualized antihypertensive management may also aid in the early detection and mitigation of CHS symptoms. During balloon dilation, inflation may activate baroreceptors in the carotid sinus and induce reflexive hypotension. As the duration of balloon inflation increases, so does the risk of stroke (48). Concurrent carotid artery wall injury, plaque disruption, increased embolus counts, and in-stent thrombosis formation can all diminish perfusion to peripheral brain tissues, potentially increasing the probability of symptomatic stroke associated with balloon use. These findings suggest that during CAS procedures, clinicians should use balloon dilation cautiously, reducing the frequency of use or considering techniques such as low-pressure dilation to minimize vascular damage. It is noteworthy that stent type (closed-cell vs. open-cell) was ultimately not retained in the final predictive model. This finding is consistent with prior large-scale meta-analyses, which demonstrated no significant differences between the two designs in terms of 30-day rates of death, stroke, or TIA (49). Similar conclusions have also been reported in other independent studies (50). Evidence suggests that closed-cell stents may confer advantages in specific subgroups, such as patients with unstable plaques, where they have been associated with lower in-hospital rates of stroke, myocardial infarction, or death (51). These observations imply that in contemporary CAS practice, with advances in device technology and increasing operator expertise, the direct impact of stent architecture on overall periprocedural risk may have been outweighed by more clinically meaningful patient-specific factors, such as plaque morphology. In our cohort, the lack of statistical significance for stent type may reflect several factors: (1) an insufficient proportion of patients with unstable plaques to detect a measurable effect, and (2) perioperative strategies, such as the choice and systematic use of embolic protection devices and optimized pharmacological management, which may have mitigated the modest differences attributable to stent design. Future prospective studies focused on high-risk subgroups are warranted to clarify whether tailored stent selection can provide incremental clinical benefit beyond current best practices.

According to the established risk factors, a nomogram was developed to facilitate the preoperative prediction of ANEs. The model demonstrated high predictive accuracy with an AUC of 0.798 in the training cohort and 0.819 in the validation cohort. The internally validated, corrected C-index of 0.773 attests to the excellent stability of the nomogram. The calibration curve demonstrated that the predicted probabilities of ANE occurrence closely matched the actual probabilities. DCA was conducted to assess the model’s clinical utility, indicating its good clinical value. The primary advantage of this study lies in the development of an easily applicable, quantifiable prediction tool that can preoperatively estimate the risk of ANEs in individuals with symptomatic carotid stenosis undergoing CAS. This not only aids in surgical decision-making and reduces event risk but also helps identify patients requiring closer postoperative monitoring due to high risk.

We conducted stratified analyses of ANEs and found that ischemic and hemorrhagic events exhibited distinct risk profiles. Ischemic ANEs were mainly influenced by age, ulcerated plaque, and hemodynamic suppression, reflecting the role of vulnerable plaque burden and impaired cerebrovascular reserve. In contrast, hemorrhagic ANEs were largely driven by peri-procedural factors such as use of balloon dilation and BPV, with hemodynamic suppression also contributing. These findings suggest that the two event types arise from different pathophysiological mechanisms and may require differentiated perioperative management strategies. Although BPV was not included in the final nomogram, it remained an independent risk factor in the hemorrhagic subgroup, underscoring its clinical importance. Several explanations may account for its exclusion from the composite model. First, BPV appears to be more strongly associated with hemorrhagic complications, as shown in the subgroup analysis, but its effect may have been diluted when ischemic events were combined into the composite endpoint. Second, BPV is closely correlated with perioperative Hemodynamic suppression, which was retained as an independent predictor, thereby diminishing BPV’s statistical weight in the overall model. From a pathophysiological perspective, elevated BPV may exacerbate the risk of postoperative cerebral hyperperfusion and vessel rupture, thereby contributing specifically to hemorrhagic complications (52). These findings indicate that perioperative blood pressure management during CAS should not only target mean levels but also aim to minimize fluctuations to reduce hemorrhagic risk. Future large-scale studies are needed to clarify the mechanistic role of BPV and to assess whether its integration into risk prediction tools could enhance individualized stratification for hemorrhagic events. It is noteworthy that the composite model achieved AUCs of 0.798 and 0.819 in the training and validation cohorts, respectively, which remained superior to those of the stratified models. This suggests that while stratified analysis improves mechanistic interpretability, the composite model retains indispensable clinical value by providing a robust and integrative tool for perioperative risk prediction. Taken together, the two approaches are complementary: stratified analysis enhances mechanistic insight, whereas the composite model offers stable predictive performance for clinical application.

This study has several limitations. First, its retrospective nature inevitably introduces risks of information bias and confounding factors. Second, due to the limited follow-up duration, the model is not applicable for predicting events occurring beyond 1 month postoperatively. Third, as the nomogram was developed in a Southern Chinese population, its applicability to Western populations may be limited owing to differences in cardiovascular risk profiles. Although we adopted a conservative variable selection strategy (LASSO combined with Bayesian validation) and performed both internal and external validation, the event-per-variable ratio (EPV = 8) remained lower than the ideal standard, which may restrict the generalizability of the model. In addition, although bootstrap resampling and external validation confirmed the overall calibration of the model, prediction in the extremely high-risk range remained somewhat uncertain, largely due to the relatively small number of such patients. Therefore, the proposed model requires further validation in prospective, multicenter studies with larger sample sizes to confirm its robustness and clinical applicability.

## Conclusion

We developed and validated a tool that effectively predicts the risk of ANEs in patients with symptomatic carotid artery stenosis undergoing CAS at an early stage. The results confirmed that age, ulcerated plaque, hemodynamic suppression, and the use of balloon dilation are significant predictors of ANEs. This novel nomogram exhibited good accuracy and good clinical utility. Its application facilitates the preoperative identification of high-risk patients, enabling clinicians to adjust interventions to mitigate potential ANEs, thereby improving overall surgical outcomes and patient safety. Moreover, this study provides a foundation for further research in several key areas. Future studies could explore the long-term outcomes of patients identified as high-risk and the effectiveness of targeted management strategies. Additionally, research should consider the integration of this tool with other risk assessment models to develop a comprehensive approach to preoperative evaluation.

## Data Availability

The raw data supporting the conclusions of this article will be made available by the authors, without undue reservation.
