# Supplementary figures and images for: A nomogram for predicting adverse neurovascular events after carotid artery stenting in patients with symptomatic carotid stenosis
Source: Front Neurol. 2025 Oct 20;16:1648838. doi: 10.3389/fneur.2025.1648838 (PMC12580134; doi:10.3389/fneur.2025.1648838)

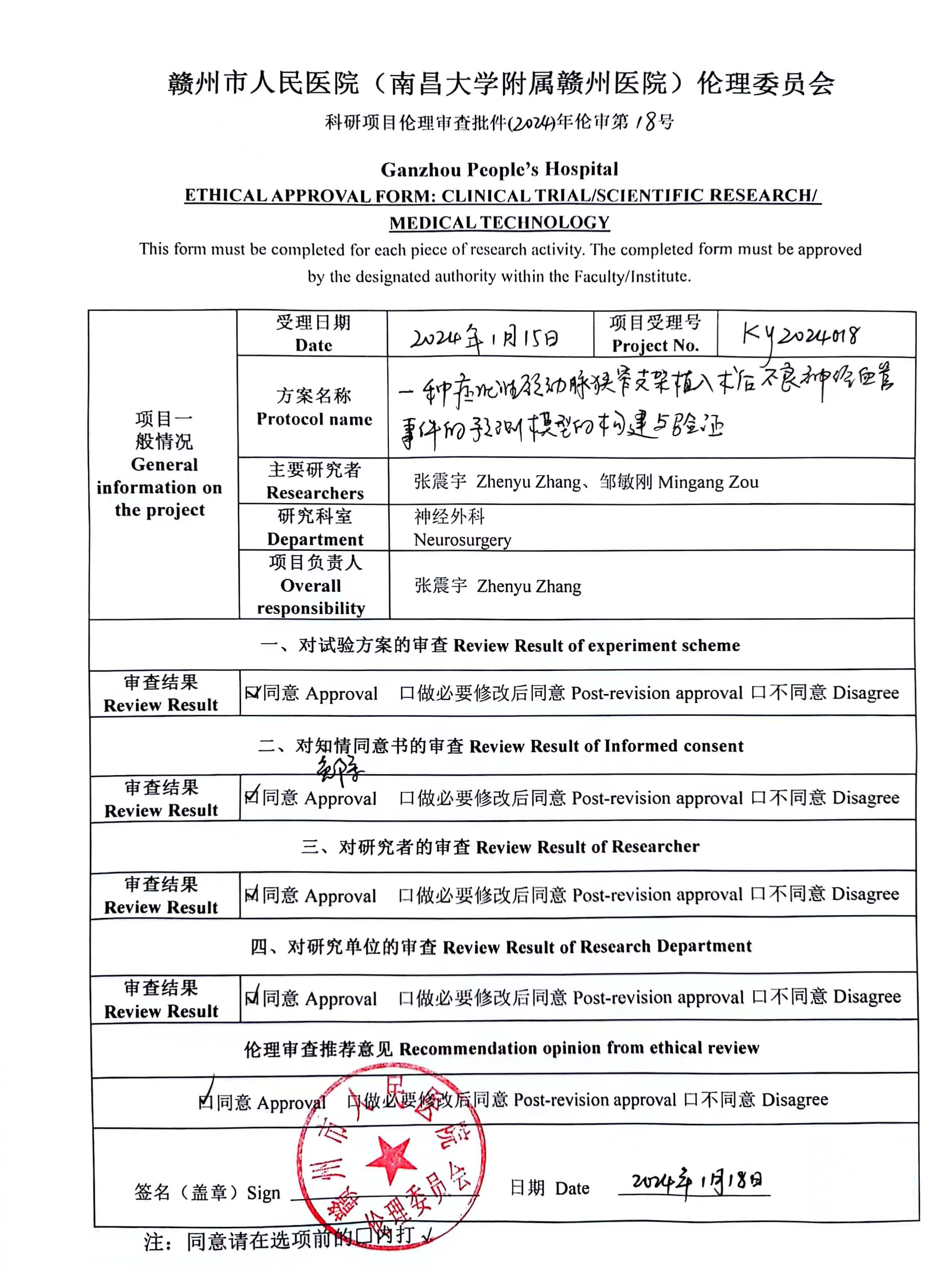

Supplement: Supplementary file 1 [file Image_1.TIFF]
