# Supplementary material for: A nomogram for predicting adverse neurovascular events after carotid artery stenting in patients with symptomatic carotid stenosis
Source: Front Neurol. 2025 Oct 20;16:1648838. doi: 10.3389/fneur.2025.1648838 (PMC12580134; doi:10.3389/fneur.2025.1648838)
Supplement: Supplementary file 2 [file Table_1.DOCX]

Supplementary Material

# Supplementary Figures and Tables

## Supplementary Figures


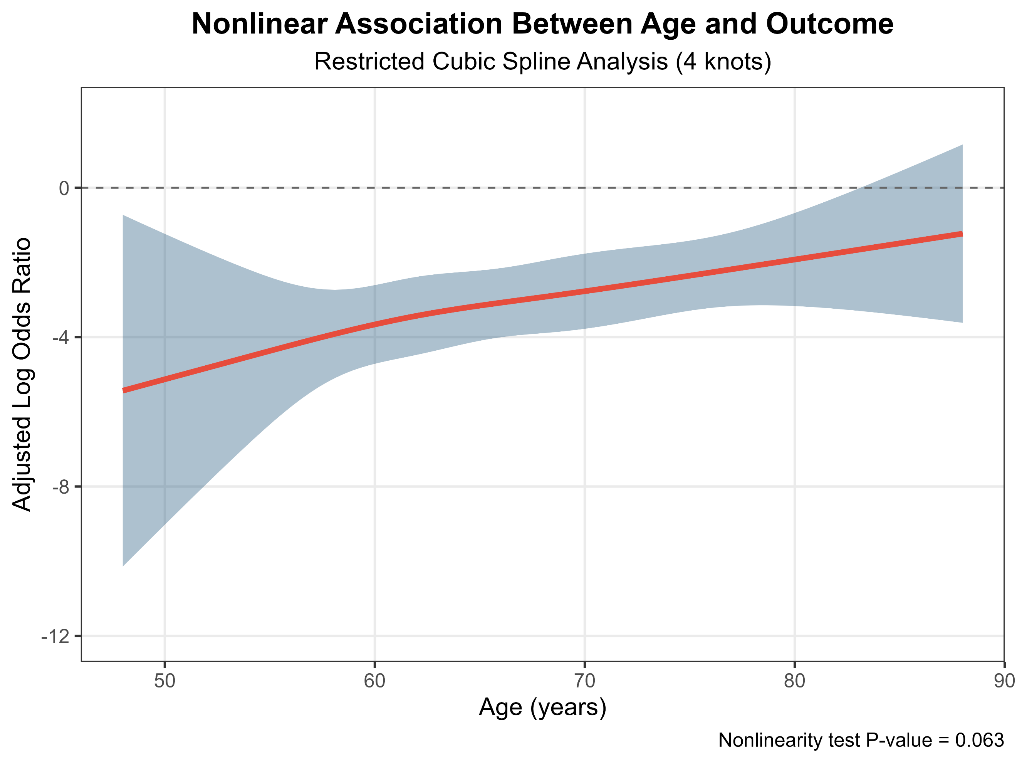


**Supplementary Figure S1** Restricted cubic spline (RCS) analysis of the nonlinear association between age and the occurrence of ANEs. The red line indicates the fitted spline curve, and the shaded blue area denotes the 95% confidence interval. The nonlinearity test yielded p=0.063, suggesting no significant departure from linearity, thus supporting the retention of age as a continuous predictor.


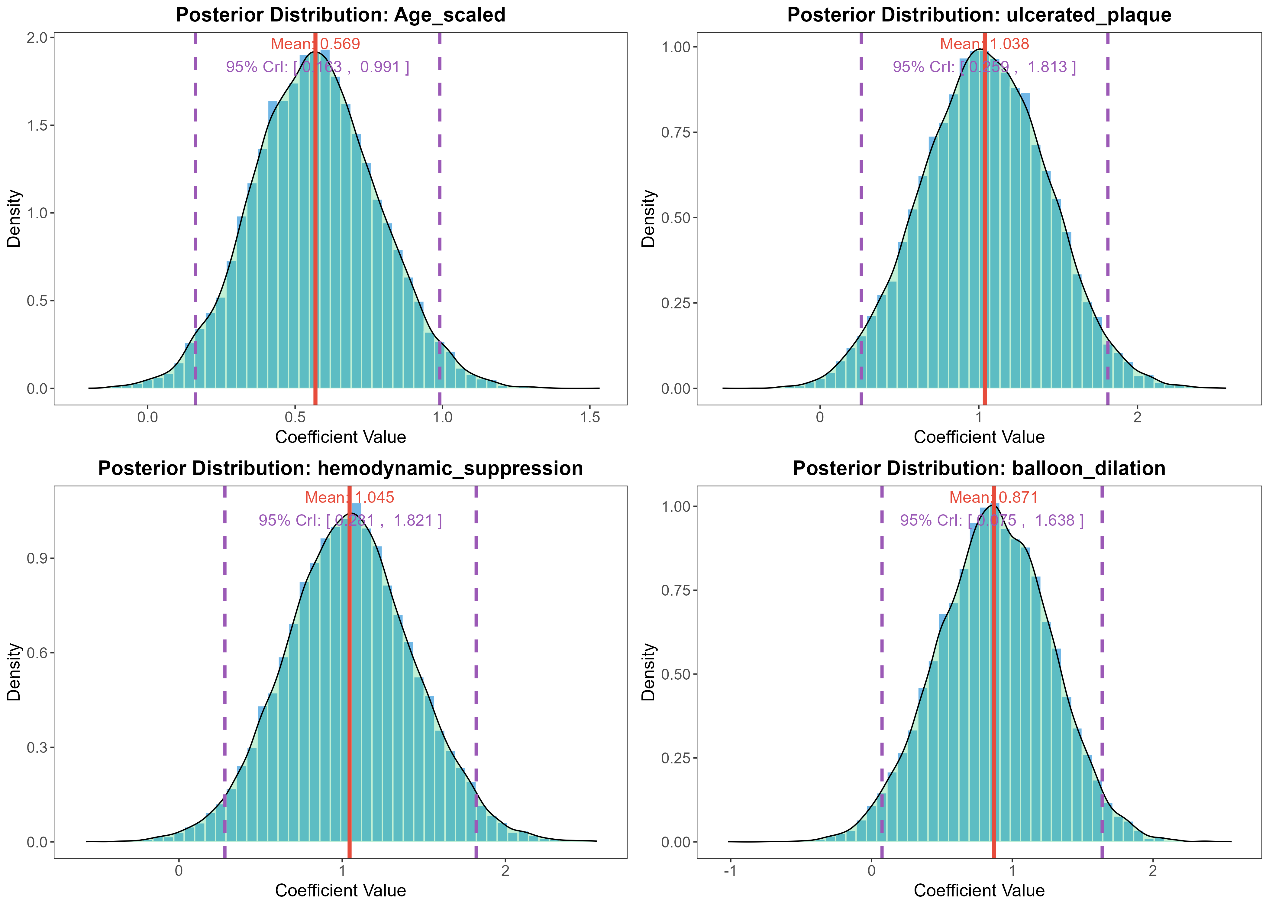


**Supplementary Figure S2**  Bayesian posterior distributions of the four predictors. All predictors exhibited unimodal distributions distinctly away from zero, indicating stable effects. The 95% credible intervals (CrI) excluded the null value, providing robust evidence of statistical significance. Posterior means quantified the most likely effect estimates, with CrIs capturing uncertainty. Collectively, these findings confirm that the predictor set is robust and the model is not overfit, even under conservative Bayesian estimation with weakly informative priors.


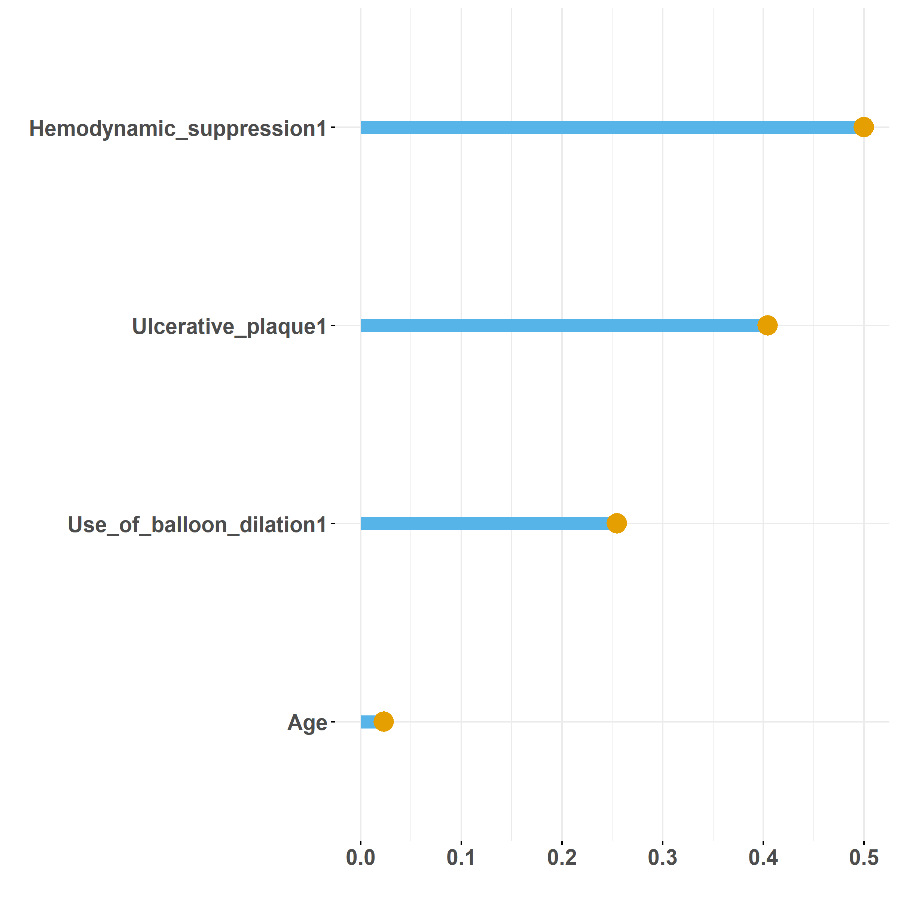


**Supplementary Figure S3**  Bar plot of standardized coefficients for the four variables selected by LASSO regression at the optimal λ (Lambda.1se). The plot illustrates both the direction and relative magnitude of each predictor’s contribution retained in the final model.


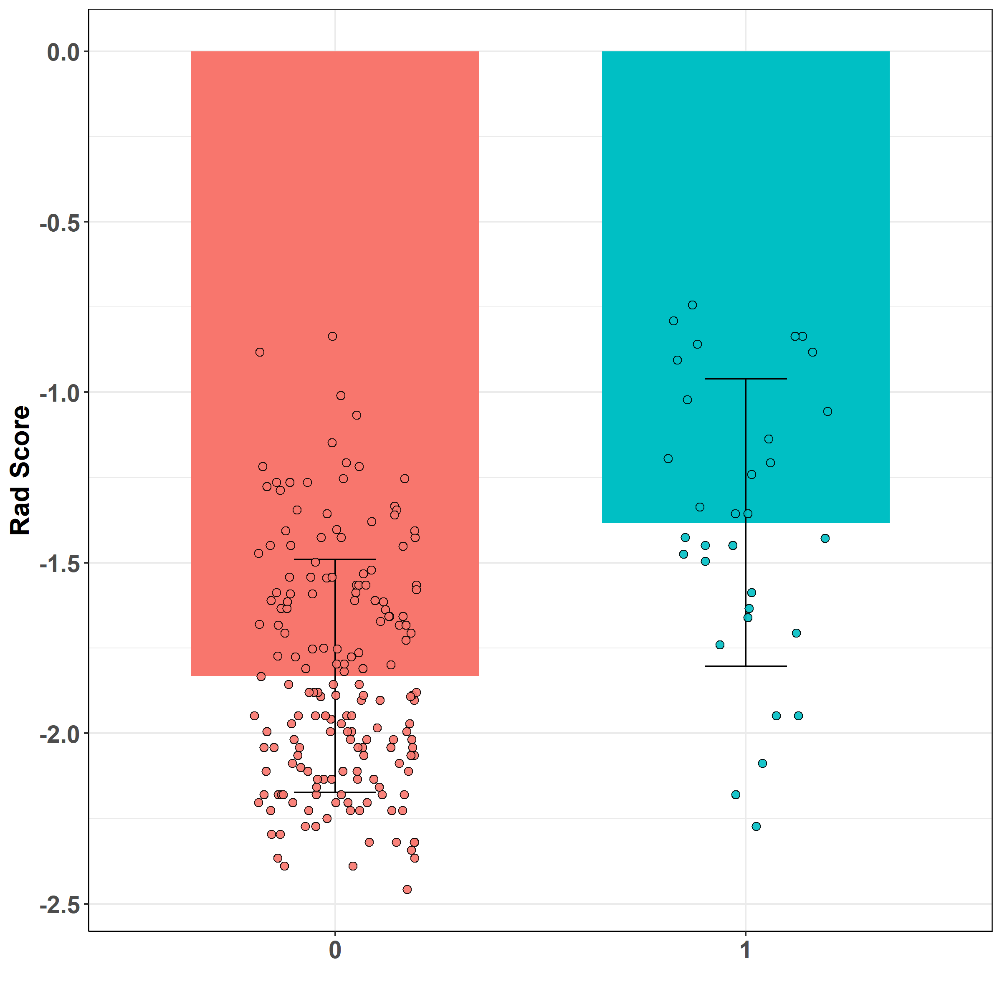


**Supplementary Figure S4**  Box plot showing the distribution of Rad scores between patients with ANEs (ANE=1) and without ANEs (ANE=0). Each dot represents an individual subject. Box limits indicate the interquartile range (IQR), horizontal lines the median, and whiskers variability outside the quartiles. Rad scores show clear separation between the two groups, highlighting the discriminatory ability of the Rad score.


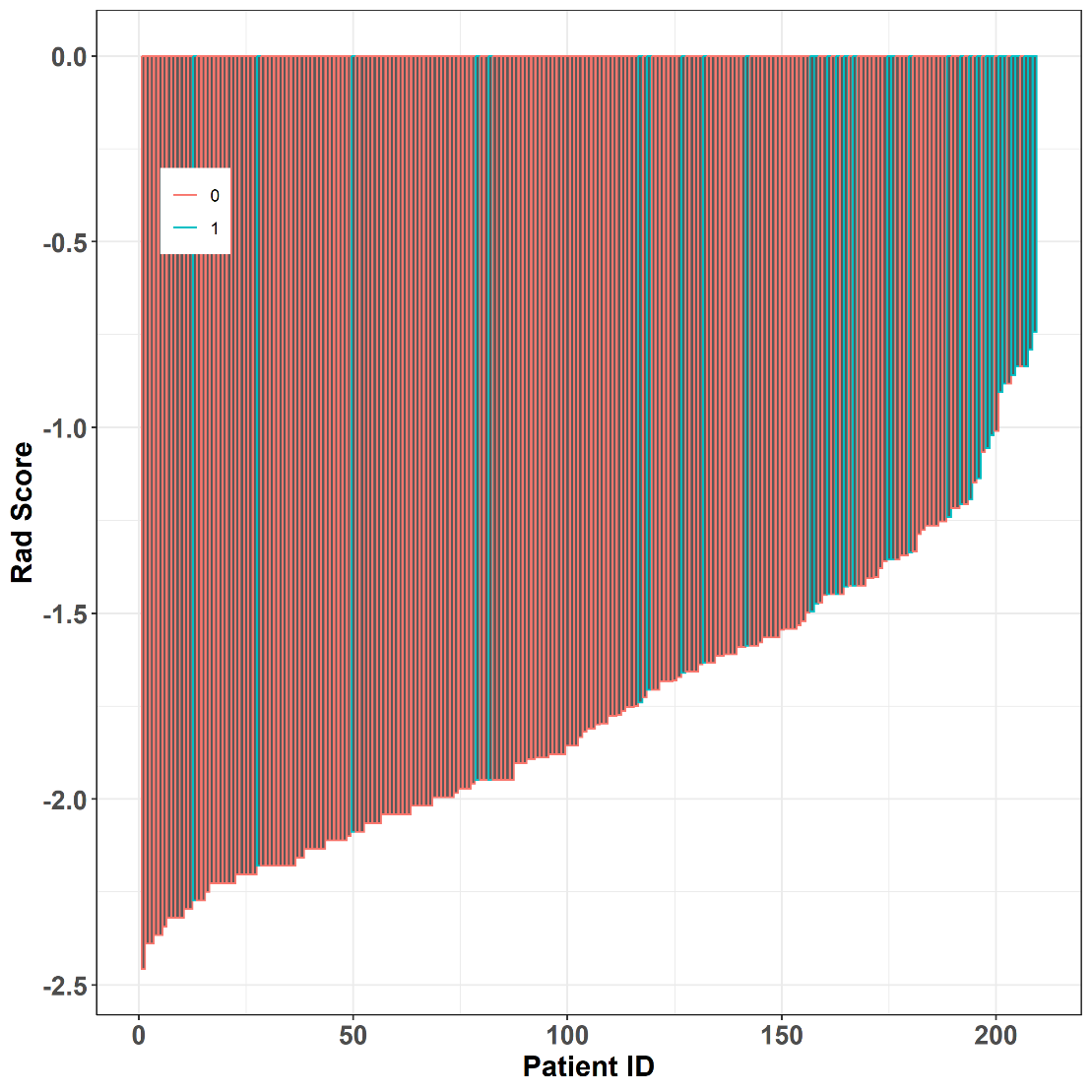


**Supplementary Figure S5** The Rad Score distribution waterfall plot demonstrates the individualized application of the model. Each bar represents a patient’s Rad Score, ordered from low to high. Although the number of event cases (ANE = 1, blue) is relatively small, the majority are concentrated in the higher Rad Score range, aligning with the expected risk stratification and highlighting the model’s discriminative capacity at the individual level.

**Supplementary Figure S6** ROC curve analysis for age as a predictor of ANEs. The optimal age cutoff was calculated as follows:

Logistic regression and ROC analysis yielded a maximum Youden index of 0.1289531, located between the values at 64 years (0.12386) and 65 years (0.13404). Using linear interpolation with *x*representing the optimal age (64 < x < 65):

​
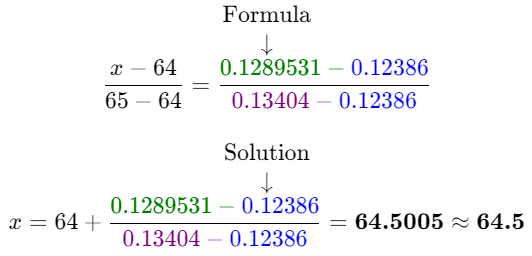


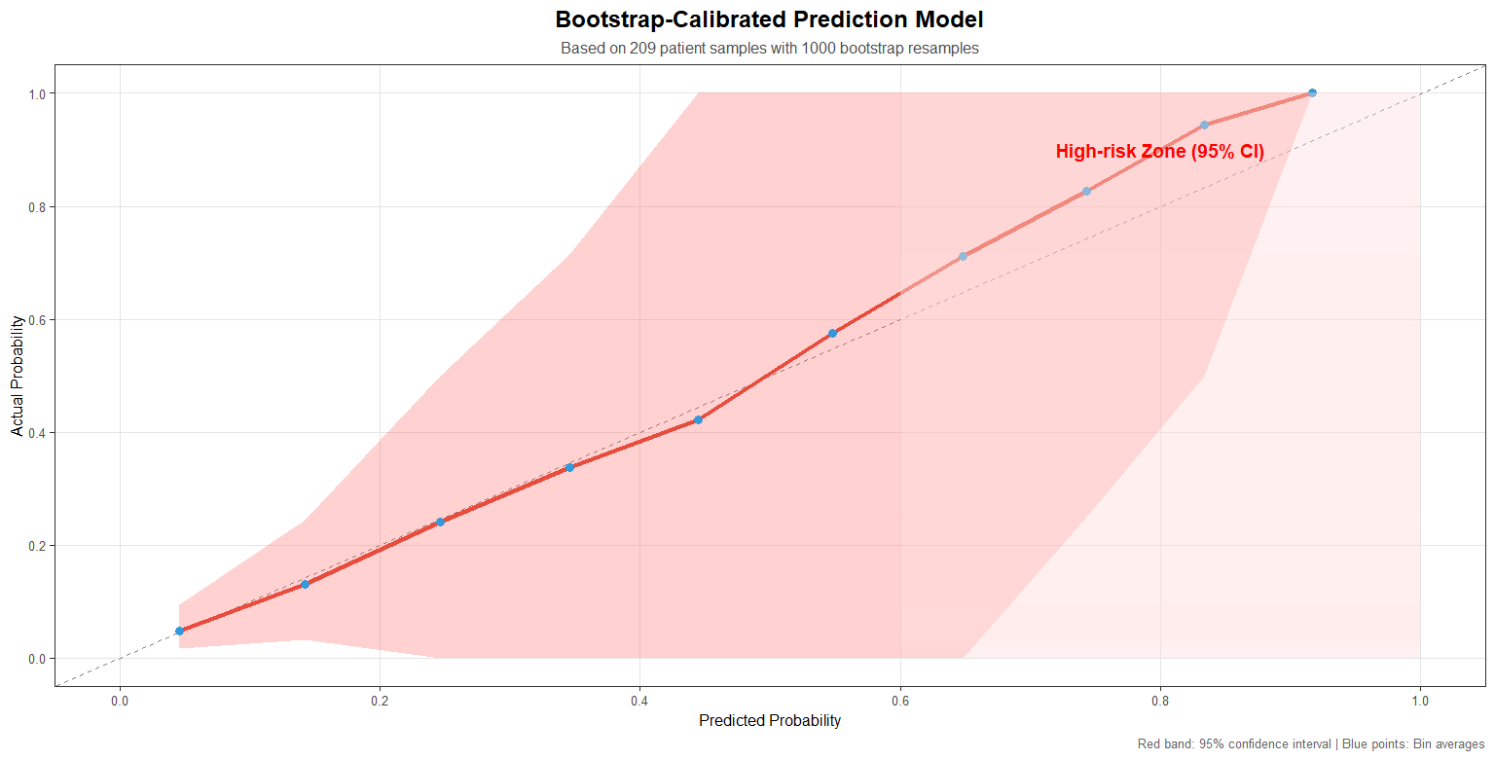

**Supplementary Figure S7** Bootstrap-corrected calibration curve for the training cohort. The calibration curve, generated with 1000 bootstrap resamples, illustrates the agreement between predicted and observed probabilities of ANEs, thereby evaluating and adjusting for potential overoptimism in the training dataset. The red line represents the bias-corrected calibration, the shaded red area indicates the 95% confidence interval, and the blue dots denote the mean predicted probabilities within each bin.

## Supplementary Tables

**Supplementary Table S1** Inter-rater agreement for TIA adjudication.

| **Cohort** | **Cohen’s κ** | **95% CI** | **Interpretation (Landis & Koch)** |
| --- | --- | --- | --- |
| Training cohort | 0.787 | 0.607 – 0.967 | Substantial agreement |
| Validation cohort | 0.736 | 0.487–0.985 | Substantial agreement |

**Supplementary Table S2** Univariate and multivariate Firth logistic regression analyses for ischemic adverse neurovascular events (ANEs) in the validation cohort.

| **Variables** | **Univariate analysis**  **( Ischemic ANEs)** | |  | **Multivariate analysis**  **(** **Ischemic ANEs )** | |
| --- | --- | --- | --- | --- | --- |
|  | **OR(95%CI)** | ***p* Value** |  | **OR(95%CI)** | ***p* Value** |
| Age | 1.140 (1.040-1.250) | 0.005 |  | 1.185 (1.050-1.370) | 0.010 |
| Ulcerated plaque | 6.067 (1.772–20.764) | 0.004 |  | 5.419 (1.172-29.691) | 0.036 |
| Hemodynamic suppression | 7.378 (1.891–28.788) | 0.004 |  | 8.953 (1.942-60.852) | 0.010 |
| Use of balloon dilation | 3.414 (1.023–11.390) | 0.046 |  | 4.047 (0.813-22.436) | 0.089 |

**Supplementary Table S3** Univariate and multivariate Firth logistic regression analyses for hemorrhagic adverse neurovascular. events (ANEs) in the training cohort.

| **Variables** | **Univariate analysis**  **( Hemorrhagic ANEs)** | |  | **Multivariate analysis**  **(** **Hemorrhagic ANEs )** | |
| --- | --- | --- | --- | --- | --- |
|  | **OR(95%CI)** | ***p* Value** |  | **OR(95%CI)** | ***p* Value** |
| Age | 1.100 (0.995–1.215) | 0.063 |  | 1.133 (0.965-1.378) | 0.151 |
| Ulcerated plaque | 4.333 (1.011–18.571) | 0.048 |  | 3.155 (0.319-35.510) | 0.316 |
| Hemodynamic suppression | 7.378 (1.424–38.238) | 0.017 |  | 8.768 (0.983-48.780) | 0.045 |
| Blood pressure variability | 1.304 (1.059–1.606) | 0.012 |  | 1.425 (1.096-2.067) | 0.023 |
| Use of balloon dilation | 9.897 (2.823–50.255) | 0.004 |  | 10.895 (3.643-59.793) | 0.008 |
